# Publisher Correction: Imagined otherness fuels blatant dehumanization of outgroups

**DOI:** 10.1038/s44271-025-00195-9

**Published:** 2025-01-28

**Authors:** Austin van Loon, Amir Goldberg, Sameer B. Srivastava

**Affiliations:** 1 Duke University, Durham, NC USA; 2 Stanford University, Stanford, CA USA; 3 University of California, Berkeley, Berkeley, CA USA

**Keywords:** Sociology, Human behaviour, Politics

Correction to: *Communications Psychology* 10.1038/s44271-024-00087-4, published online 06 May 2024

In the version of the article initially published, Figs. 4 and 5 were switched and have now been corrected in the HTML and PDF versions of the article.

