# Supplementary material for: Imagined otherness fuels blatant dehumanization of outgroups
Source: Commun Psychol. 2024 May 6;2:39. doi: 10.1038/s44271-024-00087-4 (PMC11332176; doi:10.1038/s44271-024-00087-4)
Supplement: Supplementary file 1 — Supplementary Information [file 44271_2024_87_MOESM1_ESM.pdf]

Supplementary Information

Austin van Loon[1†], Amir Goldberg[2], Sameer B. Srivastava[3]

[1]Duke University, Durham, NC, USA.
[2]Stanford University, Stanford, CA, USA.
[3]University of California, Berkeley, Berkeley, CA, USA.

[†]Corresponding author: austin.vanloon@duke.edu

Supplementary Note 1

Below, we list each of the associated concepts used in our schema elicitation task, broken out by the set in which they were presented to participants.

Supplementary Table 1 Words used in the Schema Elicitation Task

Positive Set A	Positive Set B	Negative Set A	Negative Set B	Neutral Set A	Neutral Set B
kind	healthy	unacceptable	ignorant	individualistic	collectivist
strong	important	irresponsible	irrational	artistic	pragmatic
encouraging	decent	disgusting	dangerous	competitive	cooperative
sensible	confident	stupid	hateful	traditional	modern
great	respectful	unfair	unjust	young	abstract
wise	consistent	dishonest	poor	concrete	old
sincere	truthful	destructive	foolish	urban	rural
happy	moral	selfish	awful	profit-driven	purpose-driven

Supplementary Note 2

We conducted additional analyses (some pre-registered and some exploratory) that help establish the validity of our measurement technique. Using the sample from our correlational study (described in greater detail in the main text), we first show that individuals' responses about America from their personal perspective tend to be more similar to their responses from the ingroup's perspective than to their responses from the outgroup's perspective. That is, respondents on average imagine themselves as having interpretations of America that are more similar to the prototypical ingroup member than to the prototypical outgroup member. We also show that the degree to which one imagines oneself as having interpretations of America that are more similar to one's ingroup than to one's outgroup is significantly and positively associated with one's strength of group identification. Finally, we demonstrate that outgroup divergence—that is, the distance between one's personal and outgroup schemas—is negatively related to the warmth of one's feelings about the outgroup.

Our first set of analyses tests whether individuals' ingroup schemas as measured by our instrument tend to be more similar to their personal schemas than are their outgroup schemas. We expect ingroup schemas to be more closely aligned with personal schemas than are outgroup schemas. The second assesses whether the degree to which this is the case correlates with participants' self-reported strength of ingroup identification. We anticipate that the alignment of ingroup schemas with personal schemas increases with ingroup identification. Finally, we evaluate whether outgroup divergence—that is, the distance between the personal schema and the outgroup schema—is negatively related to outgroup warmth. We predict that people who see the outgroup schema as diverging significantly from their personal schema will report feeling more negatively about their outgroup.

The figure below shows the perceived divergence of respondents' ingroup and outgroup schemas from their personal schemas, measured using the measure defined in equation (1), where A is one's personal schema and B is their ingroup and outgroup schema respectively. As can be seen—in accordance with our expectations and our pre-registration—the responses participants attributed the prototypical ingroup member were much more similar to their own responses than were the responses they attributed to the prototypical outgroup member (Cohen's $d = 1.02$). This difference between these distributions is significant according to a paired sample t-test ($t = 22.64$, $p < 0.001$).

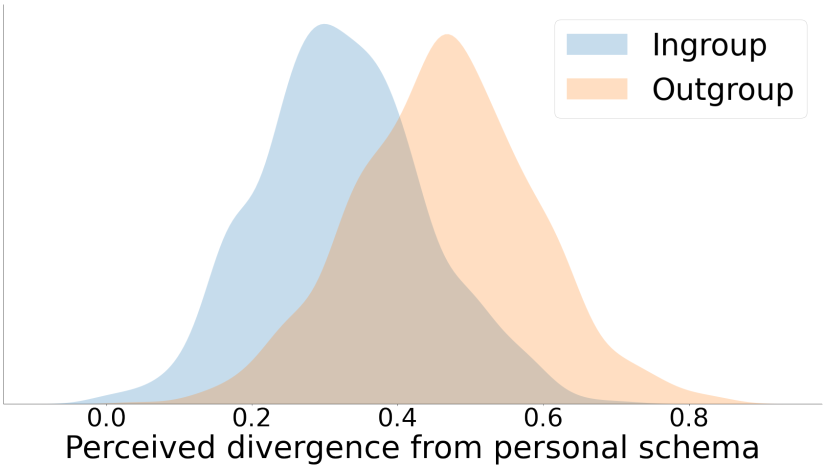

Supplementary Figure 1 Validation A. Distributions of the divergence between respondents' ingroup (blue) and outgroup (orange) schemas from their personal schemas.

For each participant, we quantify how much more similar their ingroup schema is to their personal schema than is their outgroup schema, which we term their relative intergroup divergence. We define this as the divergence between their personal and outgroup schemas (see equation 1) less the divergence between their personal and ingroup schemas. This metric is positive for participants whose ingroup schema is more similar to their personal schema than is their outgroup schema.

We test whether this is associated with strength of identity in two ways. The first is by assessing whether this is significantly correlated with participants' scores on the reduced four-item version of the partisan identity scale, which asks questions like "To what extent do you think of yourself as being a [Democrat/Republican]?" on a four-point scale. Responses to the four items were combined into a single index ($\alpha = 0.87$). Consistent with our expectations, these measures were significantly correlated according to both a Spearman ($\rho = 0.16$, $p < 0.001$) and Pearson ($r = 0.15$, $p < 0.001$) correlation analysis. The second is by evaluating whether those who self-identified as strong partisans have higher values of this measure. Indeed they did, and significantly so according to an independent samples t-test ($t = 14.02$, $p < 0.001$) and a Wilcoxon rank-sum test ($U = 6.26$, $p < 0.001$). The figure below

shows mean levels of participants' relative intergroup divergence among strong and weak Democrats and Republicans.

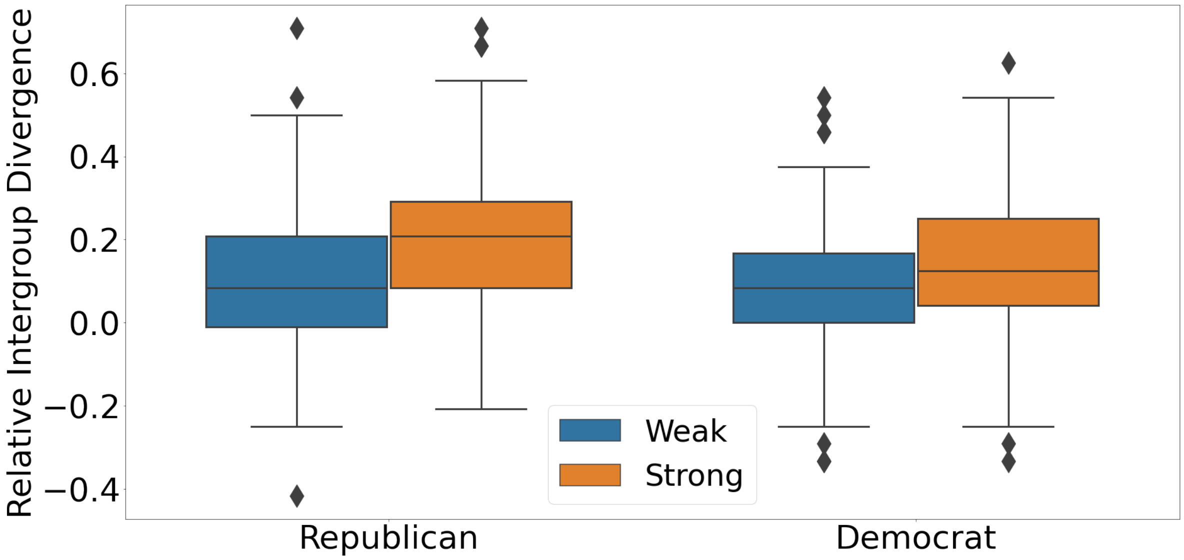

Supplementary Figure 2 Validation B. Levels of relative intergroup divergence for Democrats and Republicans who self-identified as a Strong [Democrat/Republican] (orange) and as a Not very strong [Democrat/Republican] (blue).

The table below reports results of our final validation check: assessing whether participants' outgroup divergence (i.e., the distance between their personal and outgroup schemas) is negatively related to their reported level of outgroup warmth. In Model 1, we find strong support for our predicted association. Model 2 demonstrates that this relationship holds even when we control for participants' party affiliation, the strength of their party identification, and their ideological extremity.

Supplementary Table 2 Personal-Outgroup Interpretive Divergence and Outgroup Warmth

	(1)	(2)
Outgroup Divergence	-7.65***	-6.44***
	(0.80)	(0.74)
Democrat		-5.68***
		(1.49)
Strong Partisan		-8.20***
		(1.74)
Ideological Extremity		-6.30***
		(1.00)
Constant	27.58***	46.79***
	(0.80)	(1.82)
N	772	772
R^2	0.11	0.26

"Outgroup Divergence" is standardized
Standard errors in parentheses
* $p < 0.05$, ** $p < 0.01$, *** $p < 0.001$
