# Supplementary material for: Imagined otherness fuels blatant dehumanization of outgroups
Source: Commun Psychol. 2024 May 6;2:39. doi: 10.1038/s44271-024-00087-4 (PMC11332176; doi:10.1038/s44271-024-00087-4)
Supplement: Supplementary file 3 — Reporting Summary [file 44271_2024_87_MOESM3_ESM.pdf]

nature portfolio

Corresponding author(s): Austin van Loon
Last updated by author(s): 03-27-2024

Reporting Summary

Nature Portfolio wishes to improve the reproducibility of the work that we publish. This form provides structure for consistency and transparency in reporting. For further information on Nature Portfolio policies, see our Editorial Policies and the Editorial Policy Checklist.

Statistics

For all statistical analyses, confirm that the following items are present in the figure legend, table legend, main text, or Methods section.

n/a	Confirmed	
☐	☒	The exact sample size (*n*) for each experimental group/condition, given as a discrete number and unit of measurement
☐	☒	A statement on whether measurements were taken from distinct samples or whether the same sample was measured repeatedly
☐	☒	The statistical test(s) used AND whether they are one- or two-sided<br>*Only common tests should be described solely by name; describe more complex techniques in the Methods section.*
☐	☒	A description of all covariates tested
☒	☐	A description of any assumptions or corrections, such as tests of normality and adjustment for multiple comparisons
☐	☒	A full description of the statistical parameters including central tendency (e.g. means) or other basic estimates (e.g. regression coefficient) AND variation (e.g. standard deviation) or associated estimates of uncertainty (e.g. confidence intervals)
☐	☒	For null hypothesis testing, the test statistic (e.g. *F*, *t*, *r*) with confidence intervals, effect sizes, degrees of freedom and *P* value noted<br>*Give P values as exact values whenever suitable.*
☒	☐	For Bayesian analysis, information on the choice of priors and Markov chain Monte Carlo settings
☒	☐	For hierarchical and complex designs, identification of the appropriate level for tests and full reporting of outcomes
☐	☒	Estimates of effect sizes (e.g. Cohen's *d*, Pearson's *r*), indicating how they were calculated

Our web collection on statistics for biologists contains articles on many of the points above.

Software and code

Policy information about availability of computer code

Data collection	We used Qualtrics to collect all of the data used for both studies.
Data analysis	We used Python 3.8.5 to clean, analyze, and vizualize the data. To do this, we made use of the following packages: SciPy (version 1.7.3), Pandas (version 1.5.1), Statsmodels (version 0.13.2), MatplotLib (version 3.6.2), and Seaborn (version 0.11.0).

For manuscripts utilizing custom algorithms or software that are central to the research but not yet described in published literature, software must be made available to editors and reviewers. We strongly encourage code deposition in a community repository (e.g. GitHub). See the Nature Portfolio guidelines for submitting code & software for further information.

Data

Policy information about availability of data

All manuscripts must include a data availability statement. This statement should provide the following information, where applicable:

- Accession codes, unique identifiers, or web links for publicly available datasets
- A description of any restrictions on data availability
- For clinical datasets or third party data, please ensure that the statement adheres to our policy

The data and analysis code necessary to replicate all results in this paper is available at the first author's GitHub.

Human research participants

Policy information about studies involving human research participants and Sex and Gender in Research.

Reporting on sex and gender	For the correlational study, gender was used in the sampling of participants. Specifically,we asked Prolific to provide a representative sample along various demographic dimensions including gender (51% identified as female). Gender was collected passively from Prolific after data collection. Participant gender was not collected during the experimental study.
Population characteristics	See below
Recruitment	In both studies, participants were recruited from Prolific. The use of this convenience sample may have biased our results in several ways.
Ethics oversight	Stanford IRB approved both studies.

Note that full information on the approval of the study protocol must also be provided in the manuscript.

Field-specific reporting

Please select the one below that is the best fit for your research. If you are not sure, read the appropriate sections before making your selection.

☐ Life sciences ☒ Behavioural & social sciences ☐ Ecological, evolutionary & environmental sciences

For a reference copy of the document with all sections, see nature.com/documents/nr-reporting-summary-flat.pdf

Behavioural & social sciences study design

All studies must disclose on these points even when the disclosure is negative.

Study description	The data used in this study are quantitative. The first study uses cross-sectional observational data, while the second uses data from a survey experiment.
Research sample	The research sample was collected from Prolific for both studies for reasons of convenience and cost. We sought to recruit only Democrats and Republicans. Both samples are "representative" based on a set of demographic quotas, but they are certainly unobserved characteristics on which this sample is not representative of the population.
Sampling strategy	We used Prolific's "nationally representative" sampling strategy. Sample sizes were selected on the basis of several rounds of pilot data.
Data collection	Participants completed the studies on whichever devices they were using when they signed up for the study on Prolific. Responses were recorded via Qualtrics.
Timing	The data for study 1 was collected between 2/2/2021 and 2/10/2021 The data for study 2 was collected between 4/5/2022 and 4/26/2022.
Data exclusions	For study 1, participants were dropped if they did not complete the survey (N = 40), if they took less than half the median duration to complete the study (N = 84), if they did not identify as a Democrat or Republican (N = 2). All criteria were preregistered. In study 2, participants were dropped if they did not identify as Democrat or Republican (N = 29) or if they did not give consent for us to use their data after being debriefed (N = 3).
Non-participation	In study 1, 31 participants (around 3%) did not complete the survey after starting it. In study 2, 5 participants (about 1%) did not complete the online survey after starting it. We have no way of knowing why these participants attritted.
Randomization	In study 1, there was no randomization into conditions. In study 2, participants were randomly assigned into one of two conditions by way of a Qualtrics "Randomizer" in the survey flow.

Reporting for specific materials, systems and methods

We require information from authors about some types of materials, experimental systems and methods used in many studies. Here, indicate whether each material, system or method listed is relevant to your study. If you are not sure if a list item applies to your research, read the appropriate section before selecting a response.

Materials & experimental systems

n/a	Involved in the study
☒	☐ Antibodies
☒	☐ Eukaryotic cell lines
☒	☐ Palaeontology and archaeology
☒	☐ Animals and other organisms
☒	☐ Clinical data
☒	☐ Dual use research of concern

Methods

n/a	Involved in the study
☒	☐ ChIP-seq
☒	☐ Flow cytometry
☒	☐ MRI-based neuroimaging
